# Prevalence and social determinants of smoking among men in Mauritius: a cross-sectional study

**DOI:** 10.1080/16549716.2024.2367415

**Published:** 2024-06-20

**Authors:** Miguel San Sebastián, Tuomilehto Jaakko, Stefan Söderberg, Paul Zimmet, Bhushan Ori, Jaysing Heecharan, Osvaldo Fonseca-Rodríguez, Sudhirsen Kowlessur

**Affiliations:** a Department of Epidemiology and Global Health, Umeå University, Umeå, Sweden; b Population Health Unit, Finnish Institute for Health and Welfare, Helsinki, Finland; c Department of Public Health, University of Helsinki, Helsinki, Finland; d Saudi Diabetes Research Group, King Abdulaziz University, Jeddah, Saudi Arabia; e Department of International Health, National School of Public Health, Instituto de Salud Carlos III, Madrid, Spain; f Department of Public Health and Clinical Medicine, Umea university, Umea, Sweden; g Department of Diabetes, Central Clinical School, Monash University, Melbourne, Australia; h Ministry of health and Wellness, Port Louis, Mauritius; i Non-communicable Diseases, Health Promotion and Research Unit, Ministry of Health and Wellness, Port Louis, Mauritius

**Keywords:** Socioeconomic, smoking, determinants, men, Mauritius

## Abstract

**Background:**

Mauritius has implemented a range of stringent policies to control smoking and promote public health. Regular monitoring focuses on the prevalence of tobacco use, yet there is a gap in understanding its socio-economic patterns.

**Objective:**

The aim of this study was to estimate the prevalence of tobacco smoking and to identify the social determinants associated with smoking among men in Mauritius in 2021.

**Methods:**

This is a cross-sectional population-based study conducted by the Ministry of Health and Wellness during 2021. In total, 3622 individuals participated (response rate of 84.1%), of which 1663 were men (45.9%). The study mainly focused on men given the low prevalence of smoking among women. Daily smoking was the outcome and a series of sociodemographic and socioeconomic factors were included as independent variables. Prevalence ratios (PR) and their 95% confidence intervals (95% CI) were estimated to fulfill the study objective.

**Results:**

The prevalence of smoking among men was 30.4%. People in the 25–34 age group (PR = 1.65; 95% CI: 1.12–2.41), those separated, divorced or widowed (PR = 1.57; 95% CI: 1.16–2.11), the ethnic groups Muslim-Mauritians (PR = 1.70; 95% CI: 1.00–2.89) and Creoles (PR = 1.97; 95% CI: 1.16–3.35), and those with secondary (PR = 1.29; 95% CI: 1.00–1.67) and primary education (PR = 1.47; 95% CI: 1.10–1.98) were statistically significantly associated with daily smoking.

**Conclusions:**

Although a gradual decline in smoking prevalence was observed compared with the previous 2015 survey, the Ministry of Health and Wellness should persist in fortifying its anti-smoking measures and concentrate on crafting tailored interventions aimed at the vulnerable groups identified in this study.

## Paper context

- **Main findings:** This study found a prevalence of smoking of 30.4% among men in Mauritius, identifying the young population, those not married, the Muslim-Mauritians and Creole ethnic groups and those with secondary and primary education as at-risk groups for smoking.
- **Added knowledge:** The study provides updated information on the prevalence of smoking and its distribution among different socioeconomic groups in Mauritius.
- **Global health impact for policy and action:** The anti-smoking policies implemented by the Ministry of Health and Wellness should continue to be strengthened, and specific interventions for the identified at-risk groups be developed. This can serve as a model for other countries with similar socio-economic profiles, aiming to reduce smoking consumption.

## Background

Smoking is a significant risk factor for various health problems globally. It is a leading cause of preventable deaths and is responsible for a wide range of diseases, including several cancers, heart disease, chronic obstructive pulmonary disease (COPD), stroke and type 2 diabetes [1,2]. Although the prevalence of smoking globally has decreased significantly since 1990 among both men (27.5% reduction) and women (37.7% reduction) aged 15 years and older, population growth has led to a significant increase in the total number of smokers from 0.99 billion in 1990 to 1.1 billion in 2019, with tobacco smoking still causing 7.7 million deaths – including 1 in 5 deaths in men worldwide [3].

Internationally, the prevalence of smoking varies across countries, with higher frequencies in low- and middle-income nations than high-income countries, associated with aggressive marketing tactics by tobacco industry and less stringent regulations in these countries [4–6]. By regions, the lowest average prevalence, for both men and women, has been estimated to be in the African continent, at around 18% in 2000 and 10% in 2020 [7]. However, differences exist among countries with five countries (Madagascar, Lesotho, South Africa, Seychelles and Mauritius) experiencing an age-standardized prevalence of smoking in both sexes higher than 20% [8]. A recent study pooling information from 10 countries in East Africa reported a prevalence of smoking among men of 11.0% [9].

Mauritius has implemented a range of stringent policies to control smoking and promote public health since the ratification of the World Health Organization Framework on Tobacco Control in 2004. Recognizing the detrimental effects of tobacco use on its population, the Government has taken several proactive measures to curb smoking and protect its citizens from the harms of tobacco smoking [10]. One of the key steps taken by Mauritius has been the implementation of a comprehensive ban on smoking in public places, including all indoor areas, such as restaurants, bars, and workplaces, as well as certain outdoor spaces, including beaches, sports places and public parks. To complement the smoking ban, Mauritius has also implemented strict regulations on tobacco advertising, promotion, and sponsorship by tobacco industry. These measures restrict tobacco companies from marketing their products through various channels, such as television, radio, print media, and the Internet. In 2022, the Ministry of Health and Wellness published the new Public Health regulations (Restriction on Tobacco Products) including further restrictions on the sale, distribution and promotion of tobacco products and expanding smoke-free spaces [11,12].

Despite these measures, information from the last national Non-communicable Disease Survey conducted in 2015 in the population aged 18–74 indicated still a high prevalence of smoking in the total population (19.7%) with a predominately consumption among men (38.5%) while in women smoking was rare (4.1%) [13]. While the prevalence of tobacco smoking is routinely monitored, less is known about differences among social determinants of smoking in Mauritius. The aim of this study was to estimate the prevalence of tobacco smoking and to identify the sociodemographic and socioeconomic factors associated with smoking in a representative population sample of Mauritius men in 2021.

## Methods

### Study design

Mauritius is a country in the Indian Ocean with a population of 1.3 million people, and comprises different ethnicities, including Sino-, Hindu- and Muslim- and African- (Creole) Mauritians. Non-communicable disease risk factor surveys have been repeatedly conducted since 1987 by the Ministry of Health and Wellness in collaboration with several international research institutions [13]. This study is based on the data from the latest survey in 2021.

The target population for the 2021 survey comprised all Mauritian adults aged 20–74 years. Because of the ethnic heterogeneity of the Mauritian population and to present reliable estimates of the disease and risk factor distribution, participants were drawn from all over the island. Mauritius is divided into nine districts. The sample drawn from each district was proportional to the population size of the district. Within each district, several primary sampling units (PSUs) were created and a total of 11 PSUs were randomly selected for the whole island. Two additional PSUs were selected in the district of Port Louis (Chinatown and Plaine Verte) to ensure that all ethnic groups were adequately represented, giving a final number of 13 PSUs.

After the selection of the PSUs, a complete listing of members of households was prepared. Based on estimated prevalence rates and desired levels of precision, a sample of approximately 4300 participants was chosen.

Participants were invited to survey sites in October–November 2021, and well-trained data collectors from the Ministry of Health and Wellness interviewed the participants. The completeness of the data collection was double checked at the end of the survey visit.

The survey included in addition to sociodemographic variables, a comprehensive questionnaire related to the presence of and risk factors for diabetes and cardiovascular diseases. Of 4305 people invited to participate in the survey, 3622 responded (overall response rate of 84.1%), of which 1663 (45.9%) were men.

### Measures

Smoking, the outcome for this study, was coded based on the answer to the question: ‘Do you smoke cigarettes, cigar or pipe?’ with possible answers as never, ex-smoker and currently smoker; the first two answers were coded as non-smokers.

#### Dependent variables

Four variables were included as sociodemographic factors: age was divided into six groups (<25 years, 25–34, 35–44, 45–54, 55–64 and those with 65 years and above); marital status was coded into single, married and separated/divorced/widow(er); ethnicity into Hindu-Mauritian, Muslim-Mauritian, Creole and Sino-Mauritian according to their background of origin commonly used in the country; and place of residence was defined as living in urban and rural areas.

Five more variables were included as socioeconomic factors: education was divided into primary (including those without formal school education due to the low number), secondary and tertiary/diploma; occupation was classified into professionals, associated professionals and traders, clericals, manual, housewives, retired and other (including students and unemployed); self-reported average household income (including pensions) was divided into five groups; up to 10,000 rupees (Rs; 1 euro = 47 Rs), from Rs 10,001–20,000, from Rs 20,001–35,000, from Rs 35,001–50,000 and above Rs 50,000 (those who answered ‘Don´t know’ were excluded; *n* = 129); cash margin was captured with the question of whether the participants or their household, within 1 month, manage to pay an unexpected expense of around Rs 50,000 without borrowing or asking for help? with ‘Yes/No’ as possible answers, and, finally difficulties of managing regular expenses during the last 12 months such as food, rent, bills (difficulties to make ends meet) was dichotomized into ‘Yes’ and ‘No’.

### Data analysis

Frequency tables and percentages were used to present the descriptive characteristics of the population as well as its prevalence by smoking. Prevalence ratios (PR) were used as the measure of effect using a log-binomial distribution with their 95% confidence intervals (95% CI) to provide inference. Since a preliminary analysis showed a very low prevalence of smoking among women (2.7%), the primary analysis has been conducted only among men. Analyses of the total sample are presented in supplement 1. Due to a strong correlation between age and occupation (variance inflation factor > 5), two separate models were conducted including each one of these two variables. All regression models were adjusted for the clustered nature of the observations and the potential correlation within the PSUs. All analyses were conducted with the R v 4.4.0 statistical software.

## Results

Table 1 shows the population characteristics of the sample. Out of the respondents (*n* = 1663), 25.2% were in age group 55–64 and most were married (75.1%). The most common ethnic group was the Hindu-Mauritian (58.3%) and most participants were living in rural areas (62.7%). Regarding socioeconomic status, more than half had finished secondary school (54.2%) and manual workers (33.5%) followed by retired (18.7%) were the most common occupational groups. Around half of the population (49.6%) was classified as poor or very poor, 56.2% reported not to have cash margin and almost one-fifth (19.7%) had difficulties to make ends meet.

**Table 1.** Sociodemographic characteristics of the sub-sample of men, Mauritius 2021.

Characteristic	N = 1,663
age6.f	
<25	86 (5.2%)
25–35	215 (12.9%)
35-<45	328 (19.7%)
45-<55	368 (22.1%)
55-<65	419 (25.2%)
65+	247 (14.9%)
Marital status	
Married	1,239 (75.1%)
Single	353 (21.4%)
Sep/Divorced	42 (2.5%)
Widowed	17 (1.0%)
Ethnic group	
Sino-Mauritian	90 (5.4%)
Hindu-Mauritian	969 (58.3%)
Muslim-Mauritian	356 (21.4%)
Creole	248 (14.9%)
Residence	
Rural	1,042 (62.7%)
Urban	621 (37.3%)
Education	
Tertiary	248 (15.2%)
Secondary	885 (54.2%)
Primary	501 (30.7%)
Occupation	
Professionals	182 (11.2%)
Associate professionals+Traders	325 (20.0%)
Manual workers	546 (33.5%)
Clerical	167 (10.3%)
Retired	305 (18.7%)
Students/Unemployed	104 (6.4%)
Income	
Richest	154 (9.7%)
Richer	194 (12.2%)
Middle	454 (28.5%)
Poor	623 (39.2%)
Poorest	166 (10.4%)
Cash margin	
Yes	715 (43.8%)
No	917 (56.2%)
Difficulties make ends meet	
No difficulties	1,301 (80.3%)
Yes, difficulties	320 (19.7%)

The prevalence of smoking in the men sample was 30.4% (15.4% in the total sample including both sexes). A prevalence higher than 40% was observed among men aged 25–34 years, and those who were separated/divorced/widow-er (Table 2). All the included independent variables, except place of residence, were statistically significantly associated with smoking in the univariable model. After adjustments, the 25–34 age group (PR = 1.65; 95% CI: 1.12–2.41), those separated, divorced or widowed (PR = 1.57; 95% CI: 1.16–2.11), the ethnic groups Muslim-Mauritians (PR = 1.70; 95% CI: 1.00–2.89) and Creoles (PR = 1.97; 95% CI: 1.16–3.35), and those with secondary (PR = 1.29; 95% CI: 1.00–1.67) and primary education (PR = 1.47; 95% CI: 1.10–1.98) still showed a statistically significantly higher prevalence of smoking.

**Table 2.** Crude and adjusted prevalence ratios of the relationship between the social factors and smoking (95% confidence intervals in brackets) in men, Mauritius 2021.

	Smoking N (%)	PR crude (95% CI)	PR adjusted (95%CI) (occupation excluded)	PR adjusted (95%CI) (age excluded)
Age				
<25	27 (31.8)	1	1	
25-<35	101 (47.9)	1.51 (1.07–2.12)	1.65 (1.12–2.41)	
35-<45	118 (36.5)	1.15 (0.82–1.62)	1.12 (0.75–1.68)	
45-<55	110 (30.3)	0.95 (0.67–1.35)	0.90 (0.59–1.36)	
55-<65	108 (26.2)	0.83 (0.58–1.17)	0.78 (0.51–1.20)	
65+	33 (13.6)	0.43 (0.27–0.67)	0.39 (0.23–0.66)	
Marital status				
Married	336 (27.5)	1	1	1
Single	133 (38.0)	1.38 (1.17–1.62)	1.04 (0.85–1.27)	1.34 (1.12–1.60)
Sep/Divorced/Widow-er	27 (45.8)	1.66 (1.24–2.23)	1.57 (1.16–2.11)	1.58 (1.17–2.13)
Ethnicity				
Sino-Mauritian	15 (16.9)	1	1	1
Hindu-Mauritian	274 (28.7)	1.70 (1.06–2.73)	1.33 (0.80–2.21)	1.55 (0.93–2.59)
Muslim-Mauritian	114 (32.6)	1.93 (1.19–3.14)	1.45 (0.86–2.44)	1.70 (1.00–2.89)
Creole	94 (38.5)	2.29 (1.40–3.73)	1.64 (0.97–2.76)	1.97 (1.16–3.35)
Education				
Tertiary	60 (24.2)	1	1	1
Secondary	277 (31.3)	1.29 (1.02–1.65)	1.29 (1.00–1.67)	1.09 (0.81–1.46)
Primary	159 (31.7)	1.31 (1.02–1.69)	1.47 (1.10–1.98)	1.09 (0.79–1.50)
Occupation				
Professionals	45 (24.7)	1		1
Assoc Prof. + Traders	94 (28.9)	1.17 (0.86–1.59)		0.99 (0.69–1.43)
Manual workers	204 (37.4)	1.51 (1.15–1.99)		1.25 (0.88–1.78)
Clerical	53 (31.7)	1.28 (0.92–1.80)		1.15 (0.78–1.69)
Retired	63 (20.7)	0.84 (0.60–1.17)		0.74 (0.50–1.10)
Students/Unemployed	37 (35.6)	1.44 (1.00–2.07)		1.05 (0.68–1.61)
Income				
Richest	34 (22.1)	1	1	1
Richer	57 (29.4)	1.33 (0.92–1.92)	1.18 (0.82–1.72)	1.24 (0.85–1.83)
Middle	116 (25.6)	1.16 (0.83–1.62)	1.03 (0.72–1.46)	1.01 (0.71–1.46)
Poor	219 (35.2)	1.59 (1.16–2.18)	1.34 (0.95–1.89)	1.28 (0.89–1.84)
Poorest	52 (31.3)	1.42 (0.98–2.06)	1.34 (0.90–1.99)	1.22 (0.81–1.85)
Financial strain				
Yes	190 (26.6)	1	1	1
No	305 (33.3)	1.25 (1.07–1.46)	1.12 (0.95–1.31)	1.13 (0.96–1.33)
Difficulties make ends meet				
No difficulties	371 (28.5)	1	1	1
Yes, difficulties	118 (36.9)	1.29 (1.09–1.53)	1.07 (0.90–1.28)	1.16 (0.97–1.39)
Residence				
Rural	313 (30.6)	1		
Urban	184 (30.0)	0.98 (0.84–1.14)		

Similar results were found in the total sample where men, the age group 25–34, those separated/divorced/widow, the Muslim-Mauritians and the Creole as well as manual workers were associated with smoking (Supplement 1).

## Discussion

The prevalence of smoking among men was 30.4%. To be young, single or separated/divorced/widow-er, belonging to the Muslim-Mauritians and Creole ethnic groups and to have completed secondary and primary education were relevant factors associated with daily smoking.

When comparing with the previous national survey, a decrease in the prevalence of smoking among men from 38.0% in 2015 to 30.4% in 2021 has been observed [13]. This finding also confirms previous decreasing trends in smoking nationally [14], pointing to the effectiveness of the anti-tobacco policies (taxes to tobacco products, advertisement ban, sales restrictions, labelling of cigarette packs, and health promotion campaigns) implemented in the country since the late 1980s [14,15].

A report based on the same data as this study estimated an age-adjusted prevalence of 35.3% among men and 3.7% among women [16]. Though men prevalence can be considered high compared to other countries in the SSA region, it is similar to the reported levels in Botswana (36.3%), South Africa (35.4%) and Zimbabwe (34.4%) [9].

Very few women smoked (2.7%) in this Mauritian population. As stated in the literature, the observed gender difference in smoking can be attributed to various cultural, social and economic interlinked factors. Firstly, traditional gender roles and cultural norms in Mauritius are often associating smoking with masculinity, creating a social expectation for men to smoke as a symbol of maturity or adulthood [17–19]. Additionally, advertisements and media portrayals have often targeted men, reinforcing the idea that smoking is a masculine behaviour [17].

The finding that marriage is a protective factor towards smoking was also expected since this relationship is well known in the literature [20,21]. The beneficial effect of marriage has been postulated to work by offering more social support and control of health behaviours, particularly among men [22].

The higher prevalence of smoking among the Muslim-Mauritians and Creole ethnic groups was not a surprise, either. Mauritius is a diverse and multicultural society with a complex history of colonization, migration, and the blending of various ethnic and cultural groups. Ethnic discrimination, particularly towards the Creoles, has been an issue in Mauritius, often intersecting with linguistic, socio-economic, and historical factors [23,24]. Extensive literature globally directs to the connection between perceived racial or ethnic discrimination and health outcomes and behaviours, being smoking one of these adverse consequences [25,26].

There was an important gap in smoking between those with tertiary education (24.2%) and the other educational groups (31%). Low education has been associated with increased smoking prevalence as individuals with lower levels of education often have limited awareness of the health risks associated with smoking. They may also face barriers to accessing smoking cessation resources and exhibit greater susceptibility to tobacco marketing tactics. Moreover, socioeconomic factors linked to lower education levels, such as stress and limited financial resources, can contribute to smoking initiation and maintenance [27].

While not statistically significant, manual workers presented the highest prevalence of smoking among the occupational groups. Occupation is one distinctive social factor commonly associated with smoking where lower social class workers tend to smoke more due to a combination of social, economic, psychological, and environmental factors [28,29]. Lower social class workers often face financial stress, job insecurity, and economic difficulties, which can lead to smoking as a coping mechanism [27]. These circumstances are probably involved in the high prevalence of smoking found among manual workers in our study.

Interestingly other classical socioeconomic factors associated to smoking such as place of residence (rural/urban) and income were not relevant in this study population [30,31]. This lack of association might partly be explained by the existent correlation between manual workers social class and income.

## Methodological considerations

The large sample size together with the high response rate and the inclusion of an ample geographical and ethnic diversity are important strengths of this study that contribute to its internal and external validity.

There are however some considerations that should be taken into account when interpreting the results. Participants in population-based studies may not be representative of the entire population due to self-selection or non-response bias, which can skew the results and limit the generalizability of the findings to the broader population. However, the systematic sampling strategy applied, and the high participation rate may avoid from these potential biases in this study. Additionally, while the Hindu-Mauritians was the most common ethnic group in the study sample, they also represent the highest proportion of the population in Mauritius compared to the other groups. However, sample weighting was not applied since this information was not specified in the data collection procedures, which could have slightly modified some of the estimates.

As any other population-based study, the possibility of response bias due to the self-reported nature of variables, particularly the socioeconomic variables or even the outcome, smoking, might have affected the validity of the study results. Despite numerous variables were included in the model covering a broad range of the social determinants of smoking, failure to account for some other confounding factors might have led to inaccurate estimates of the association. The extent of these biases is however impossible to determine. Given the cross-sectional design of the study, it is always challenging to pretend to establish a cause-and-effect relationship between the examined determinants and smoking behaviour.

## Conclusions

This study found a prevalence of smoking of 30.4% among men in Mauritius, identifying the young population, those not married, the Muslim-Mauritians and Creole ethnic groups and those with secondary and primary education as at-risk groups for smoking. While the observed reduction in smoking over time is encouraging, the Ministry of Health and Wellness should continue strengthening its anti-smoking policies, enhancing the inter-sectoral collaboration with other Ministries and organizations and developing specific interventions for the identified at-risk groups in order to reduce the prevalence as well as the social inequalities in smoking in the country.

## Supplementary Material

Supplemental Material

## Data Availability

Data cannot be shared publicly because of the sensitive nature. Data are available from Umeå University (contact via the correspondent author) for researchers who meet the criteria for access to confidential data.
