# Supplementary material for: Prevalence and social determinants of smoking among men in Mauritius: a cross-sectional study
Source: Glob Health Action. 2024 Jun 20;17(1):2367415. doi: 10.1080/16549716.2024.2367415 (PMC11191822; doi:10.1080/16549716.2024.2367415)
Supplement: Supplemental Material [file ZGHA_A_2367415_SM9556.docx]

**Supplement 1. Analysis conducted only on the total sample (men and women)**

Table S1. Sociodemographic characteristics of the total sample, Mauritius 2021.

| Characteristic | N = 3,622^1^ |
| --- | --- |
| Sex |  |
| Women | 1,959 (54.1%) |
| Men | 1,663 (45.9%) |
| Age |  |
| <25 | 179 (4.9%) |
| 25-35 | 472 (13.0%) |
| 35-44 | 699 (19.3%) |
| 45-54 | 795 (22.0%) |
| 55-64 | 913 (25.2%) |
| 65+ | 564 (15.6%) |
| Marital status |  |
| Single | 645 (17.9%) |
| Married | 2,706 (75.1%) |
| Separate/ Divorced/Widow-er | 251 (6.9%) |
| Ethnic group |  |
| Hindu-Mauritian | 2,101 (58.0%) |
| Muslim-Mauritian | 780 (21.5%) |
| Creole | 537 (14.8%) |
| Sino-Mauritian | 204 (5.6%) |
| Residence |  |
| Rural | 2,299 (63.5%) |
| Urban | 1,323 (36.5%) |
| Education |  |
| Tertiary | 457 (12.8%) |
| Secondary | 1,923 (53.9%) |
| Primary | 1,190 (33.3%) |
| Occupation |  |
| Professionals | 349 (9.8%) |
| Associated professionals+Traders | 475 (13.3%) |
| Clerical | 427 (12.0%) |
| Manual workers | 875 (24.5%) |
| Housewives | 600 (16.8%) |
| Students/Unemployed | 231 (6.5%) |
| Retired | 609 (17.1%) |
| Income |  |
| Richest | 248 (7.2%) |
| Richer | 345 (10.1%) |
| Middle | 939 (27.3%) |
| Poor | 1,360 (39.6%) |
| Poorest | 542 (15.8%) |
| Cash margin |  |
| Yes | 1,406 (39.5%) |
| No | 2,157 (60.5%) |
| Difficulties make ends meet |  |
| No difficulties | 2,706 (76.6%) |
| Yes, difficulties | 829 (23.5%) |
| ^1^n (%) | |

Table S2. Crude and adjusted prevalence ratios (PR) of the association between the social factors and smoking (95% confidence intervals in brackets), Mauritius 2021.

|  | Smoking  N (%) | PR crude (95% CI) | PR adjusted (95% CI)  (occupation excluded) | PR adjusted (95% CI)  (age excluded) |
| --- | --- | --- | --- | --- |
| Sex |  |  |  |  |
| Women | 53 (2.7) | 1 | 1 | 1 |
| Men | 497 (30.4) | 11.11 (6.58 – 18.77) | 11.36 (6.78-19.04) | 9.52 (6.16-14.72) |
| Age |  |  |  |  |
| <25 | 31 (17.5) | 1 | 1 |  |
| 25-34 | 118 (25.4) | 1.45 (1.05-2.01) | 1.49 (1.13-1.96) |  |
| 35-44 | 136 (19.8) | 1.13 (0.84-1.51) | 1.16 (0.91-1.47) |  |
| 45-54 | 116 (14.7) | 0.84 (0.58-1.22) | 0.86 (0.62-1.18) |  |
| 55-64 | 114 (12.6) | 0.72 (0.54-0.97) | 0.77 (0.56-1.04) |  |
| 65 + | 35 (6.3) | 0.36 (0.23-0.57) | 0.39 (0.25-0.59) |  |
| Marital status |  |  |  |  |
| Married | 367 (13.7) | 1 | 1 | 1 |
| Single | 146 (22.8) | 1.67 (1.36-2.04) | 1.08 (0.88-1.31) | 1.36 (1.09-1.70) |
| Separated/Divorced/Widow-er | 36 (14.5) | 1.05 (0.71-1.56) | 1.83 (1.49-2.26) | 1.73 (1.33-2.25) |
| Ethnicity |  |  |  |  |
| Sino-Mauritian | 18 (8.9) | 1 | 1 | 1 |
| Hindu-Mauritian | 285 (13.7) | 1.54 (1.03-2.30) | 1.25 (0.98-1.59) | 1.34 (1.00-1.78) |
| Muslim-Mauritian | 117 (15.2) | 1.70 (1.04-2.78) | 1.41 (1.03-1.94) | 1.51 (1.03-2.20) |
| Creole | 130 (24.6) | 2.76 (1.91-3.98) | 2.19 (1.76-2.72) | 2.31 (1.72-3.09) |
| Residence |  |  |  |  |
| Rural | 345 (15.2) | 1 |  |  |
| Urban | 205 (15.6) | 1.02 (0.78-1.33) |  |  |
| Education |  |  |  |  |
| Tertiary | 68 (14.9) | 1 |  |  |
| Secondary | 302 (15.7) | 1.06 (0.69-1.62) |  |  |
| Primary | 179 (15.1) | 1.01 (0.70-1.47) |  |  |
| Occupation |  |  |  |  |
| Professionals | 49 (14.0) | 1 |  | 1 |
| Associated prof +Traders | 95 (20.0) | 1.42 (1.01-2.01) |  | 1.12 (0.80-1.59) |
| Clerical | 64 (15.0) | 1.07 (0.74-1.55) |  | 1.35 (0.92-1.97) |
| Manual workers | 222 (25.4) | 1.81 (1.23-2.65) |  | 1.51 (0.99-2.31) |
| Housewives | 10 (1.7) | 0.12 (0.05-0.29) |  | 0.63 (0.30-1.32) |
| Students/Unemployed | 45 (19.5) | 1.39 (1.00-1.92) |  | 1.37 (0.87-2.16) |
| Retired | 64 (10.5) | 0.75 (0.49-1.15) |  | 0.79 (0.52-1.21) |
| Income |  |  |  |  |
| Richest | 34 (13.7) | 1 |  |  |
| Richer | 62 (18.0) | 1.31 (0.99-1.75) |  |  |
| Middle | 128 (13.6) | 0.99 (0.70-1.42) |  |  |
| Poor | 237 (17.4) | 1.27 (0.90-1.79) |  |  |
| Poorest | 69 (12.7) | 0.93 (0.65-1.34) |  |  |
| Cash margin |  |  |  |  |
| Yes | 203 (14.4) | 1 |  |  |
| No | 345 (16.0) | 1.11 (0.89-1.37) |  |  |
| Difficulties make ends meet |  |  |  |  |
| No difficulties | 399 (14.7) | 1 |  |  |
| Yes, difficulties | 143 (17.3) | 1.17 (0.92-1.50) |  |  |
